# Cardiogenic shock accompanied by dynamic left ventricular outflow tract obstruction and myocardial bridging after transient complete atrioventricular block mimicking ST-elevation myocardial infarction: a case report

**DOI:** 10.1186/1756-0500-6-478

**Published:** 2013-11-19

**Authors:** Seonghui Kang, Sanghee An, Hyung Min Yu, Jiwan Kim, Sung Hea Kim, Hyun-Joong Kim, Sang Man Chung

**Affiliations:** 1 Department of Internal Medicine, Konkuk University School of Medicine, Seoul, Korea; 2 Department of cardiology, Konkuk University School of Medicine, Seoul, Korea

**Keywords:** STEMI, Dynamic LVOT obstruction, Transient MR, Myocardial bridge, Complete AV block

## Abstract

**Background:**

Dynamic left ventricular outflow tract obstruction with or without mitral regurgitation is typically observed in hypertrophic cardiomyopathy, but is also occasionally seen without left ventricular hypertrophy. In this report, we present a case of cardiogenic shock that mimics ST-elevation myocardial infarction, due to dynamic left ventricular outflow tract obstruction with transient mitral regurgitation and myocardial bridging after transient complete atrioventricular block.

**Case presentation:**

A 65-year-old man with hypertension presented himself at the emergency department with syncope after chest pain. His initial electrocardiography showed inferior ST elevation with profound precordial ST depression and transient complete atrioventricular block. Due to sustained hypotension, an intra-aortic balloon pump was applied. His coronary angiography revealed almost normal right coronary artery and left circumflex artery and only a severe myocardial bridge in the mid-segment of his left anterior descending artery. Instead, severe mitral regurgitation was found without regional wall motion abnormality both in the left ventriculography and the portable echocardiography. However the severe mitral regurgitation completely disappeared in follow up echocardiography the day after. The pressure gradient across the left ventricular outflow tract was measured at 8.95 mmHg during the resting state, and was increased to 38.95 mmHg during the Valsalva state.

**Conclusions:**

The patient presented with a case of cardiogenic shock that mimicked ST-elevation myocardial infarction due to dynamic left ventricular outflow tract obstruction combined with myocardial bridging in the mid-left anterior descending artery.

## Background

Dynamic left ventricular outflow tract (LVOT) obstruction with or without mitral regurgitation is typically observed in hypertrophic cardiomyopathy, but is also seen in other diseases, including acute coronary syndrome [1], stress-induced cardiomyopathy [2] and left ventricular hypertrophy (LVH) [3]. Recent studies reported that it may occur even in the absence of LVH and cause symptoms such as chest pain, syncope or both [4,5].

Myocardial bridging is defined as a segment of a major epicardial coronary artery, the ‘tunneled artery,’ that goes intramullary through the myocardium beneath the muscle bridge [6]. The majority of such abnormalities were found in the left anterior descending coronary artery, whereas those that were found in the left circumflex (LCx) or the right coronary artery (RCA) were rare [7]. The estimated frequency of their discovery varies from 0.5% to 16% when assessed via coronary angiography [8,9]; however in some autopsy series, it is as high as 85.7% [10]. Most clinical findings have shown that the myocardial bridge does not lead to major problems; however some cases have been reported of unstable perfusion of the coronary arteries that caused serious cardiac disorders such as myocardial ischemia, myocardial infarction, arrhythmia and even sudden cardiac death. In fact, several clinical studies have shown that myocardial bridging occasionally causes acute myocardial infarction [11-14]. In this report, a case is presented of cardiogenic shock that mimicked ST-elevation myocardial infarction (STEMI) due to dynamic LVOT obstruction combined with myocardial bridging in the mid-left anterior descending artery (LAD) after transient complete atrioventricular block (AV block).

## Case presentation

**Figure 1:**
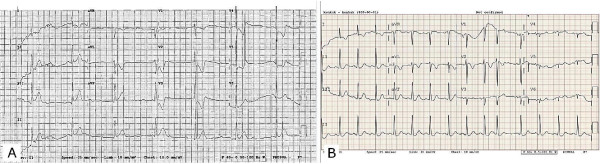
**Electrocardiography (A)** Initial electrocardiography shows complete atrioventricular block and posterior and inferior myocardial infarction. **(B)** After three minutes, the complete atrioventricular block and ST-segment changes disappeared.

**Figure 2:**
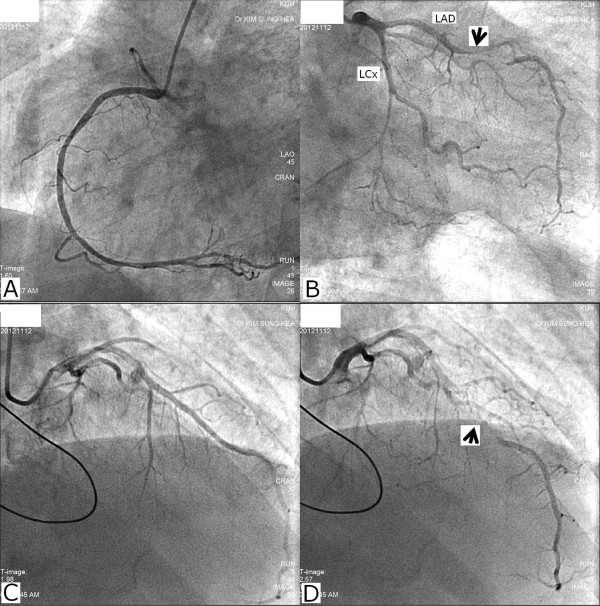
**Coronary angiography (A)** Right coronary artery (right anterior oblique view) **(B)** There are diffuse mild atherosclerotic lesions on hypoplastic distal left circumflex artery but thrombolysis in myocardial infarction (TIMI) 3 flow was noticed. Myocardial bridge was identified on mid-left anterior descending artery (black errow) **(C) (D)** Severe dynamic obstruction due to myocardial bridge (black arrow) are observed in mid segment of left anterior descending artery-wrapping apex. LAD, left anterior descending artery; RCA, right coronary artery; LCx, Left circumflex artery.

**Figure 3:**
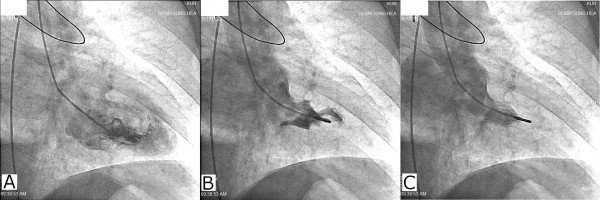
On left ventriculgraphy, mitral regurgitation of grade 3 was noticed (A,B,C).

A 65-year-old man with essential hypertension for 18 years was admitted to the emergency department due to syncope and chest pain. He had lost consciousness an hour earlier after feeling chest pain while standing at a subway station in the morning. He lost consciousness for less than five minutes. For the past four years, he had experienced syncope after chest pain once a year, but spontaneously regained consciousness. He had a 50-year history of smoking and was taking regular medications that included cilnidipine, aspirin, atenolol and hydrochlorothiazide. Upon his hospital admission, he still complained of substernal squeezing chest pain with sweating. His vital signs were 69/42 mmHg and 48 beats per minute, and his initial electrocardiography (ECG) showed complete AV block; prominent ST depression in leads I, aVL and V2-V5; and slight ST-segment elevation in leads II, III and aVF (Figure 1A). Under the impression of inferior STEMI, normal saline loading, intravenous heparin and inotropic agents were applied immediately in preparation for emergency coronary angiography. Three minutes after the initial ECG, reversed ECG was taken to check the presence of right ventricular infarct. It showed that the complete AV block and ST-segment changes, which are presented in Figure 1A, had disappeared (Figure 1B). Due to the sustained hypotension despite improved ST changes, an emergent intra-aortic balloon pump (IABP) was applied in the catheterization laboratory and the patient’s mean blood pressure was restored to over 60 mmHg. After the stabilization of the patient’s blood pressure, coronary angiography was performed. Contrary to the authors’ expectations, there was no significant intracoronary lesion in the RCA and the LCx (Figures 2A and 2B, respectively). Instead, severe dynamic obstruction due to myocardial bridging was observed in the mid-segment of the LAD-wrapping apex (Figures 2C and 2D), and Grade 3 mitral regurgitation (MR) was noticed in the left ventriculography (Figure 3).

**Figure 4:**
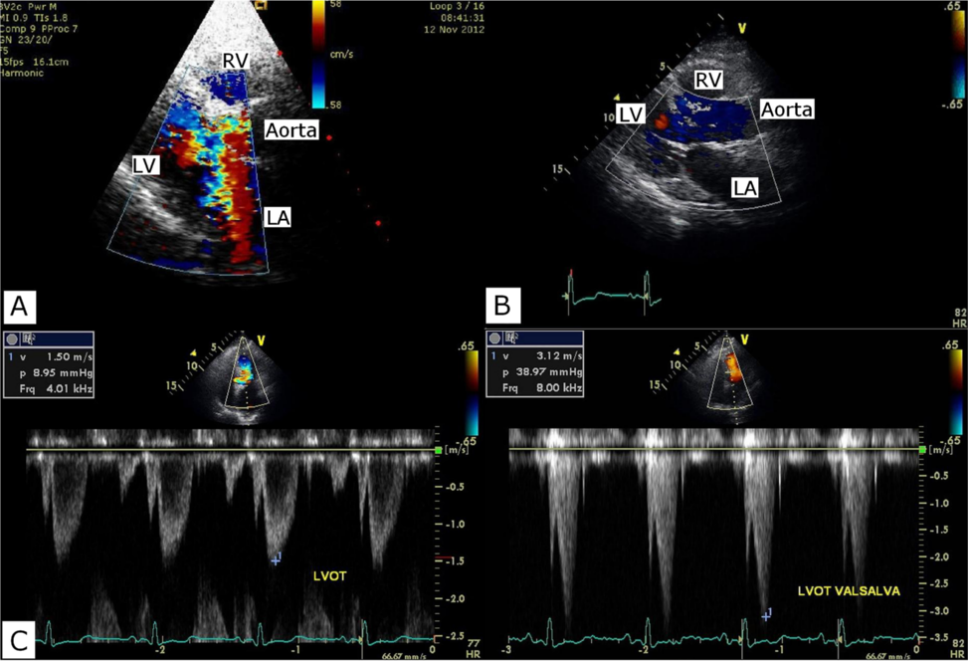
**Echocardiography (A)** Portable echocardiography that performed immediately after coronary angiography showed severe mitral regurgitation and systolic flow acceleration of left ventricular outflow tract obstruction on Hospital day 1. **(B)** On follow up echocardiogram after intra-aortic balloon pump weaning, mitral regurgitation completely disappeared. (Hospital day 3) **(C)** On follow up echocardiography after intra-aortic balloon pump weaning (Hospital Day 3), V_max_ is 1.5 m/sec and peak pressure gradient is 8.9 mmHg at resting state. At Valsalva maneuver, V_max_ is 3.1 m/sec and peak pressure gradient is 38.9 mmHg. LA, Left atrium; LV, Left ventricle; RV, Right ventricle; V_max_ , maximum velocity.

The portable echocardiography that was performed immediately after the coronary angiography showed severe mitral regurgitation and systolic flow acceleration of the LVOT (Figure 4A). However, the LVOT pressure gradients were not measured due to the MR doppler contamination. There was no regional wall motion abnormality or systolic anterior motion (SAM) of mitral leaflet.

On the first day of the patient’s hospital stay, intravenous furosemide was needed due to his newly-developed pulmonary edema. Because of the hypotension and dynamic LVOT obstruction, nitrate was not used. On the next day, the IABP was successfully removed without complication. In the follow-up echocardiography after the IABP weaning, the mitral regurgitation completely disappeared and the pressure gradient across the LVOT was restored to 8.95 mmHg during the resting state. The pressure gradient across the LVOT was increased to 38.9 mmHg during the Valsalva state (Figure 4C). There was no left ventricular hypertrophy. The peak cardiac myoglobin and troponin-I at the time of the patient’s admission were 12.6 ng/ml and 0.118 ng/ml, respectively. On the seventh day after admission, dobutamine stress echocardiography was performed to determine the degree of the dynamic LVOT obstruction between the resting and stressful states; however, there was no significant increase in the pressure after the dobutamine infusion. Moreover, there was no structural abnormality in the aortic valve or the subaortic structure in the transesophageal echocardiography. On the basis of the evaluation, the patient was treated medically with diltiazem. The prescription of a β-blocker was avoided due to the fact that the possibility of vasospasm could not be completely excluded. In addition, the hydrochlorothiazide was stopped to avoid aggravating the condition of LVOT obstruction. Indeed, the patient remained complaint-free during the first six months of follow-up.

## Discussion

This case is that of mimicked acute coronary syndrome with transient ST change, complete AV block and MR, which was presented as syncope after chest pain and was resolved after IABP insertion. However, there are still remaining debates as to the main pathology, the role of an increased LVOT pressure gradient and myocardial bridging.

Dynamic LVOT obstruction may be the main possible mechanism of cardiogenic shock rather than the intracoronary pathology. Although the initial presentation looked like a coronary artery problem in the medical history and the ECG, there was no significant atherosclerotic fixed lesion that explains the ECG change. In addition, severe mitral regurgitation, which occurs in the condition in which there is no regional wall motion abnormality, is less likely to originate from the stunned myocardium due to coronary spasm. Recent studies reported that symptomatic dynamic LVOT obstruction may occur even in patients without LVH [5,15]. Another study showed that even a small increased pressure gradient defined by a Doppler velocity > 1.5 m/s during dobutamine stress echocardiography is an independent positive predictor of chest pain and syncope [4]. Thus, neither prominent LVH nor increased pressure gradient after exercise is necessary to prove dynamic LVOT obstruction as a main pathology. Therefore, it seems reasonable that mitral regurgitation originates from dynamic LVOT obstruction in the special condition of severe hypovolemia, even if the patient has no LVH or no increased pressure gradient after exercise [15]. In addition, LVOT obstruction is a common precipitating factor in stress cardiomyopathy [16,17]. In this case, stress-induced cardiomyopathy may be excluded as a cause of mitral regurgitation because there was no takotsubo-like left ventricle (LV) change.

A spasm of myocardial bridging may affect the localization of the ECG change. The initial ECG showed a transient complete AV block rhythm, inferior ST segment elevation and prominent ST depression in the anterolateral leads (Figure 1A). These changes usually reflect inferior and posterior wall myocardial ischemia involving an AV node artery. In this patient, however, no significant coronary lesion was found in the RCA or the LCx. Accordingly, it may be attributed to the fact that a hidden atherosclerotic lesion in the RCA or the LCx in the presence of systemic hypotension may cause a more profound ECG change. Another possible explanation is subendocardial anterior wall ischemia due to myocardial bridging in the mid-segment of the LAD wrapping apex. Usually, myocardial bridging does not lead to major problems by itself. However, the presence of systemic hypotension and dynamic LVOT obstruction, which are well-known aggravators of myocardial bridging, could strongly affect myocardial bridging, which may result in profound subendocardial ischemia in the LAD territory. Another possible explanation is that the transient complete AV block may have preceded ischemic change. The transient complete AV block may have simply originated from the vasovagal reaction to the prolonged standing posture and may aggravate dynamic LVOT obstruction via developing AV dissociation and mitral regurgitation. This, in turn, would cause the ischemic change to appear on the ECG.

## Conclusion

A rapid diagnosis of STEMI is mandatory for optimal treatment. However, a small proportion of patients with suspected STEMI suffer from other conditions. In this report, a case of cardiogenic shock was presented that mimicked STEMI, due to dynamic left ventricular outflow tract obstruction with transient mitral regurgitation and myocardial bridging after transient complete AV block. It is hoped that this case could be of help to the treatment of conditions mimicking acute STEMI in patients referred for primary percutaneous coronary intervention.

## Consent

Written informed consent was obtained from the patient for publication of this Case Report and any accompanying images. A copy of the written consent is available for review by the Editor-in-Chief of this journal.

## Abbreviations

LVOT: Left ventricular outflow tract; LVH: Left ventricular hypertrophy; LCx: Left circumflex artery; RCA: Right coronary artery; STEMI: ST-elevation myocardial infarction; LAD: Left anterior descending artery; AV block: Atrioventricular block; ECG: Electrocardiography; IABP: Intra-aortic balloon pump; MR: Mitral regurgitation; SAM: Systolic anterior motion; LA: Left atrium; LV: Left ventricle; RA: Right atrium; Vmax: Maximum velocity.

## Competing interests

The authors declare that they have no competing interests.

## Authors’ contributions

SK, the first author, wrote the manuscript and made the figures. SK, the corresponding author, performed the diagnostic coronary angiography and the IABP and revised the manuscript. SC and JK performed the echocardiography. SA, HY and HK participated in the design of the report and helped draft the manuscript. All authors read and approved the final manuscript.
